# Improved access to kidney transplantation with favorable graft survival in highly sensitized candidates under the French acceptable mismatch program

**DOI:** 10.3389/ti.2026.16762

**Published:** 2026-09-08

**Authors:** Corinne Antoine, Emilie Savoye, Benoit Audry, Camille Legeai, Myriam Pastural, Anne Cesbron, Valerie Dubois, Jean-Luc Taupin

**Affiliations:** 1 Organ and Tissue Procurement and Transplantation Department, Agence de la Biomédecine, Saint-Denis, France; 2 Kidney Transplant Department, Saint-Louis Hospital, Assistance Publique - Hôpitaux de Paris, Paris, France; 3 Histocompatibility and Immunogenetics Laboratory, Nantes, France; 4 Laboratoire HLA, EFS Auvergne Rhône Alpes, Lyon, France; 5 Laboratory of Immunology and Histocompatibility, Hôpital Saint-Louis APHP, Paris, France; 6 INSERM U976 Institut de Recherche Saint-Louis, Université Paris Diderot, Paris, France

**Keywords:** acceptable mismatch program, allocation, highly sensitized, human leukocyte antigen, kidney transplantation

## Abstract

The scarcity of the human leukocyte antigen (HLA)-compatible donor pool, which leads to a long waiting time for transplantation, often requires specific national or supranational strategies to improve kidney graft access for highly sensitized (HS) patients. Acceptable national mismatch priority is a way to increase the number of potential matched donors without increasing the immunologic graft failure risk. In 2005, the French Acceptable Mismatch program (AMP) was successfully introduced as a strategy to improve kidney allocation for HS patients. The AMP was enhanced in 2015, authorizing up to four HLA-A, -B, -DR, and -DQβ mismatches, provided that each mismatch was an acceptable antigen. Our study compared graft survival between AMP allocation and other kidney allocation modalities among HS recipients between 2016 and 2022 (N = 2,985). After adjustment for donor and recipient characteristics using Cox regression, we found that the risk of graft failure was higher for HS non-AMP transplantations (hazard ratio 1.19 [95% CI 1.04–1.36]; 1.31 [95% CI 1.08–1.59] with death censored). The French AMP improves access to kidney transplantation for HS patients. Post-transplant survival is comparable to other kidney allocation modalities, possibly because of the absence of preformed donor-specific antibodies in recipients’ historical and recent serum, which is associated with reduced risk of rejection.

## Graphical Abstract

**Figure:**
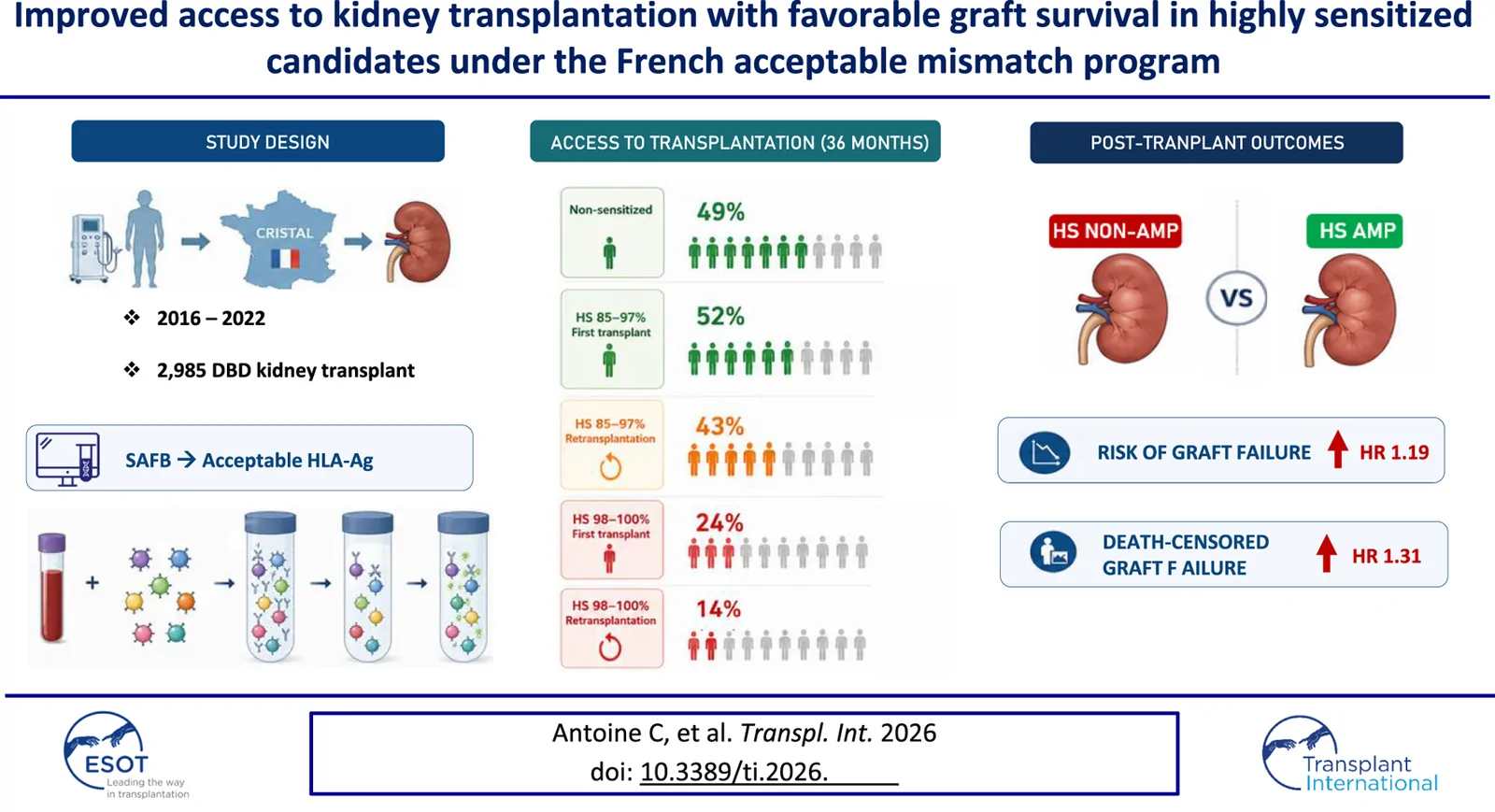

## Introduction

Highly sensitized (HS) patients, who have high levels of preformed anti-HLA (human leukocyte antigen) antibodies, face significant challenges in accessing kidney transplantation because of the limited pool of HLA-compatible donors. The definition of a candidate considered HS varies across countries and national consensus statements. Currently, two main criteria are used to assess the severity of sensitization: Virtual Panel-Reactive Antibodies^1^ and calculated Reaction Frequency (cRF)^2^. Both estimate the proportion of deceased donors incompatible with a patient owing to pre-formed HLA antibodies [1]. This immunologic barrier leads to prolonged waiting times and increased morbidity and mortality while on a waiting list.

To address this issue, several complementary strategies exist worldwide: 1) national priorities for candidates with high cRF and long dialysis duration, including Acceptable Mismatch Programs (AMPs); 2) expansion of kidney-exchange programs and cross-border sharing, both expanding donor options without increasing rejection risk or compromising long-term graft survival; 3) immunologic forgiveness policies (unacceptable antigen delisting for antibodies that declined in level or disappeared over time or that do not activate complement); and 4) desensitization strategies.

In France, national priorities in the allocation rules are based on two principles: difficulty in access to transplantation and the excellence of HLA matching. A kidney recipient is considered HS if their peak cRF is ≥85%; these patients automatically receive national HS priority, provided that their current cRF remains >70%. The system operates via three sequential, prioritized allocation pathways, with systematic age matching and ABO blood-group compatibility exemption in certain circumstances (see below). The pathways are 1) full-match HLA-A, -B, and -DR priority; 2) the AMP; and 3) a priority allowing no more than one mismatch for HLA-A, -B, and -DR between the donor and the recipient. In 2024, 9% of new registrations and 17% of active prevalent candidates were considered hyperimmunized [2].

In some cases, a long list of unacceptable HLA antigens (Ag) or high cRF is insufficient to explain immunologic difficulties in accessing a transplant. This is particularly true because the national score for allocating kidney grafts favors a good HLA match, especially for class-II Ag in recipients <45 years old. This situation may be due to rare haplotypes, including those with no linkage disequilibrium or partial or complete homozygosity. Therefore, another indicator is used: the Potential Matched Donor (PMD). The PMD estimates the number of compatible and well-HLA matched donors among all utilized kidney donations after brain determination of death (DBDD) donors within the same blood group over the past 5 years [3, 4].

A low PMD score indicates that the patient has very few suitable donors. Consequently, these patients receive additional points in the French National Kidney Allocation Score (NKAS) whenever a compatible and well-matched donor becomes available (see Table 1 for a description of kidney transplantation allocation pathways). This final point explains why a certain number of HS patients undergo transplantation via the NKAS system.

**TABLE 1:** Kidney transplantation allocation pathways for highly sensitized (HS) candidates and the approximate share of kidney transplants for each.

Allocation pathway	Approximate share	Priority level	Description
Full HLA match (A, B, DR)* ^,^ **	∼7%	Priority #1	Allocation to recipients with full HLA A, B, DR match with the donor
Acceptable mismatch program (AMP)* ^,^ **	∼47%	Priority #2	Access to grafts with each HLA mismatch considered “acceptable” based on individual antibody profiles (SAFB profile) and ≤4 HLA-A, -B, -DR, and -DQβ mismatches
≤1 HLA mismatch (A, B, DR)* ^,^ **	∼9%	Priority #3	For recipients with ≤1 mismatch among HLA-A, -B, and -DR
Score-based allocation	∼25%	Standard	Allocation according to the national scoring system that integrates waiting time, age matching, HLA matching, and sensitization factors such as PMD.
Other and combined graft	∼6%	​	​
Living donor	∼6%	​	​

* Age matching: the donor is not >15 years younger if the recipient is ≥50 years old).

** Blood group exemptions: restricted blood group exemption in group A for AB HS, candidates and in group O for B HS, candidates as soon as the cRF, reaches 85%; ABO, compatible exemption for all HS, candidates when the cRF, reaches 95%.

Remark: Non-AMP, recipients belong to Priority #1, Priority #2, and Standard Score-based Allocation.

HLA: human leukocyte antigen; PMD: potential matched donor; SAFB: single antigen flow beads.

France introduced its AMP in 2005 (Table 2). An initial evaluation in 2010 demonstrated improved access to kidney transplantation for HS patients, with no difference in graft survival from other patients. Within 2 years, access to transplantation for highly immunized incident patients increased, but access decreased for non-immunized or slightly immunized patients during the same period [5]. The AMP was significantly enhanced in 2015 with the adoption of advanced solid-phase assays, such as Luminex, which have revolutionized HLA antibody (Ab) screening and identification, particularly for class-II HLA Ag. Single Antigen Flow Bead (SAFB) assays enable precise, semi-quantitative detection of HLA Ab and acceptable HLA Ag, in accordance with national guidelines issued by the French Society of Histocompatibility and Immunogenetics (SFHI) [6].

**TABLE 2:** French acceptable mismatch program.

Eligible patient criteria
• Waiting time >18 months • Peak cRF >85%, based on results from SAFB assays; current cRF >70% • List of acceptable HLA mismatches based on both current and historical sera, with normalized MFI of less than 500, updated every 3 months in the CRISTAL database
Kidney offer conditions
• If the recipient is >50 years, the donor must not be >15 years younger than the recipient • Restricted derogation to group A for AB patients and to group O for B patients is granted once cRF reaches 85%. Derogation to group O for A and AB recipients is allowed only when cRF ≥95% • Institutional minimum match criteria must be fulfilled, especially regarding the number of HLA class-II mismatches: ≤4 HLA-A, -B, -DR, or -DQβ mismatches, ≤1 HLA-DQβ mismatch, and ≤1 HLA-DR mismatch • Each mismatch at HLA-A, B, DR, or DQβ must be acceptable • Negative pretransplant virtual crossmatch required before kidney graft transfer • Negative T and B cell crossmatch required before kidney transplantation

cRF: calculated reaction frequency; HLA: human leukocyte antigen; MFI: mean fluorescence intensity.

The « virtual cross match » is routinely implemented in all transplant centers in France to decrease the duration of cold ischemia and the risk of delayed refusal for immunologic reasons [6]. The conditions for performing a pretransplant virtual crossmatch in an emergency are standardized by the national scientific committee of the *Agence de la Biomédecine* (official referral procedure 7 June 2016, 2016G-04 deliberation). HLA-C, -DP, and -DQa typing is now available for each deceased donor, which improves the pretransplant virtual crossmatch accuracy [6].

The aim of this study was to 1) evaluate the transplant activity of HS patients and 2) compare post-transplant outcomes of HS kidney transplant recipients according to whether they benefited from the French AMP or other allocation modalities for HS transplantation.

## Materials and methods

### Study design, endpoints, and populations

For all analyses, HS recipients were defined as those with peak cRF≥ 85% and current cRF >70%. Non-sensitized recipients were defined as those with current cRF 0% or without registered HLA antibodies. Moderately sensitized (MS) recipients were defined as those with peak cRF <85% and current cRF 1%–84% or peak cRF ≥85% and current cRF ≤70%. MS recipients were divided into two groups: group A, recipients with peak cRF <85% and current cRF <85%, and group B, recipients with peak cRF ≥85% and current cRF ≤70%.

Data on the kidney waiting list and transplant activity were available for HS and MS recipients from 2016 to 2024. Additionally, longitudinal analyses of new registrations from 2005 to 2024 by transplant number, sensitization, and age were available.

For the analysis of access to transplantation, we included candidates registered on the waiting list from 2016 to 2023. Candidates were stratified by transplant number (first vs. retransplantation) and sensitization (non-sensitized, peak cRF85%–97%, and peak cRF 98%–100%). Data for MS groups A and B are available in the Supplementary Materials.

For the post-transplant survival analysis, we included all adult single-organ kidney transplant recipients from DBDDs in France from 2016 to 2022, with a mean follow-up of 45.5 months. The analysis focused on non-sensitized and HS recipients. Cox regression was restricted to HS recipients to ensure that the analysis focused on risk factors relevant to this population. HS recipients were stratified by allocation modality (AMP vs. non-AMP). In a supplementary analysis, HS non-AMP recipients were further divided into two groups: those with and without the presence of subsequently delisted preformed donor-specific antibodies (DSAs). Different survival outcomes were studied: death-censored graft loss and overall graft failure, including patient death.

For Supplementary Materials, the frequency of acute or chronic rejection frequency was calculated for HS AMP, HS non-AMP without DSA, and HS non-AMP with DSA recipients. Please note that this data entry is not exhaustive and the data quality is not audited.

Patients were followed until 1 January 2025.

### Data collection and definition of variables

The French national transplant registry (CRISTAL, *Agence de la Biomédecine*, Saint-Denis, France) prospectively collects demographic, clinical, and laboratory data for all organ transplant recipients and donors in France, as well as transplant outcomes. Data are recorded at the time of registration (inclusion in the transplant waiting list), procurement, and transplantation and then annually thereafter. Data collection is mandatory, and research technicians double-check the completeness and accuracy of data. In accordance with French law, research studies based on this national registry are part of transplant assessment and do not require additional institutional review board approval. The database has been reported to the French National Commission on Computing and Liberty.

Donor estimated glomerular filtration rate (eGFR) values were estimated with the CKD-EPI formulas [7].

### Statistical methods

Data are expressed as mean (SD, standard deviations) or median ([IQ], interquartile ranges), depending on the data distribution or number (%, percentage). For the multivariate analysis, missing data were imputed to the least risky and most frequent category when possible: body mass index (<1%, 2 recipients) imputed to the median, missing HLA-DQ mismatches (<1%, 2 transplantations) imputed to 0, and donor hypertension (<1%, 17) imputed to no. A complete-case analysis was performed as a sensitivity analysis.

Access to kidney transplantation was estimated using the cumulative incidence function, with death or delisting for worsening condition treated as competing risks, and group comparisons performed with Gray’s test. Data were censored 3 years after registration or at the time of delisting for reasons other than worsening condition or kidney transplantation, whichever occurred first.

Post-transplant survival curves were estimated with the Kaplan-Meier method and compared with the log-rank test. Factors associated with post-transplant failure were assessed with multivariable Cox proportional-hazards models. Candidate covariates for multivariable analysis were selected by an adjustment strategy that combined statistical and clinical considerations. Variables associated with the outcome at p < 0.20 in univariable analyses, along with those deemed clinically relevant based on transplant expertise and the literature, were considered for inclusion in the multivariable model. A stepwise selection procedure with a large p-value threshold (p < 0.20) was used to derive the final model while ensuring retention of clinically important covariates. The hazard ratio (HR) for the outcome with continuous variables was explored graphically with restricted cubic splines; graphs were defined according to spline plots and clinical relevance. The primary objective was to estimate the total effect of the AMP protocol on post-transplant outcomes. Consequently, we excluded variables considered potential mediators of this effect, including HLA matching, DSAs, and cold ischemia time from the adjustment set to avoid overadjustment. Adjustment for these variables could have accounted for mechanisms by which the AMP exerts its effect on outcomes. The selection of adjustment variables was guided by a prespecified causal framework, summarized in a directed acyclic graph (Supplementary Figure S1).

Statistical analyses were performed with SAS enterprise guide 9.3 (SAS Institute, Cary, NC, USA). All *p*-values were two-tailed and *p* < 0.05 was considered statistically significant.

## Results

### Kidney waiting list and transplant activity of HS recipients

**FIGURE 1:**
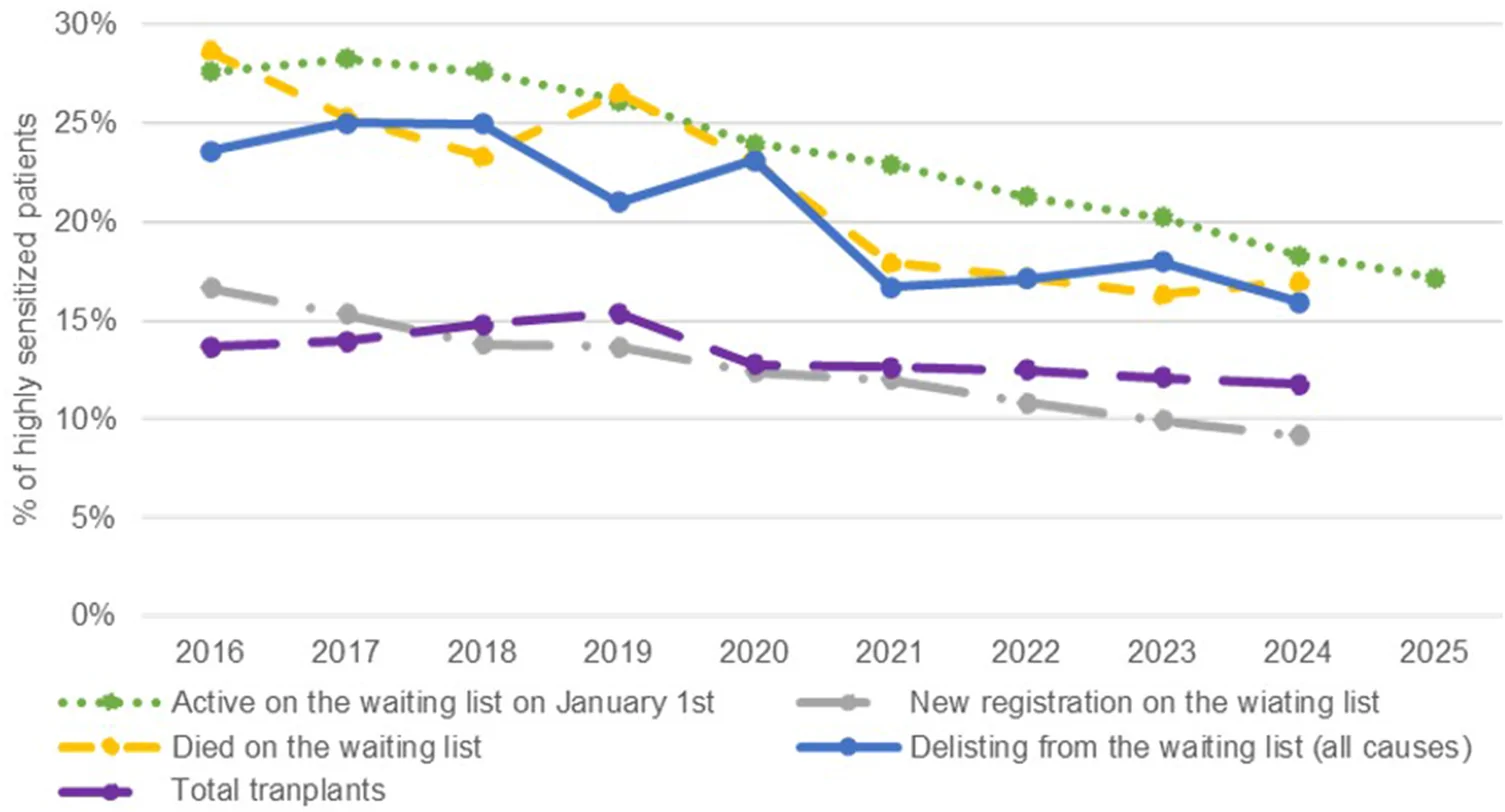
Waiting list and transplant activity of highly sensitized (HS) patients.

**FIGURE 2:**
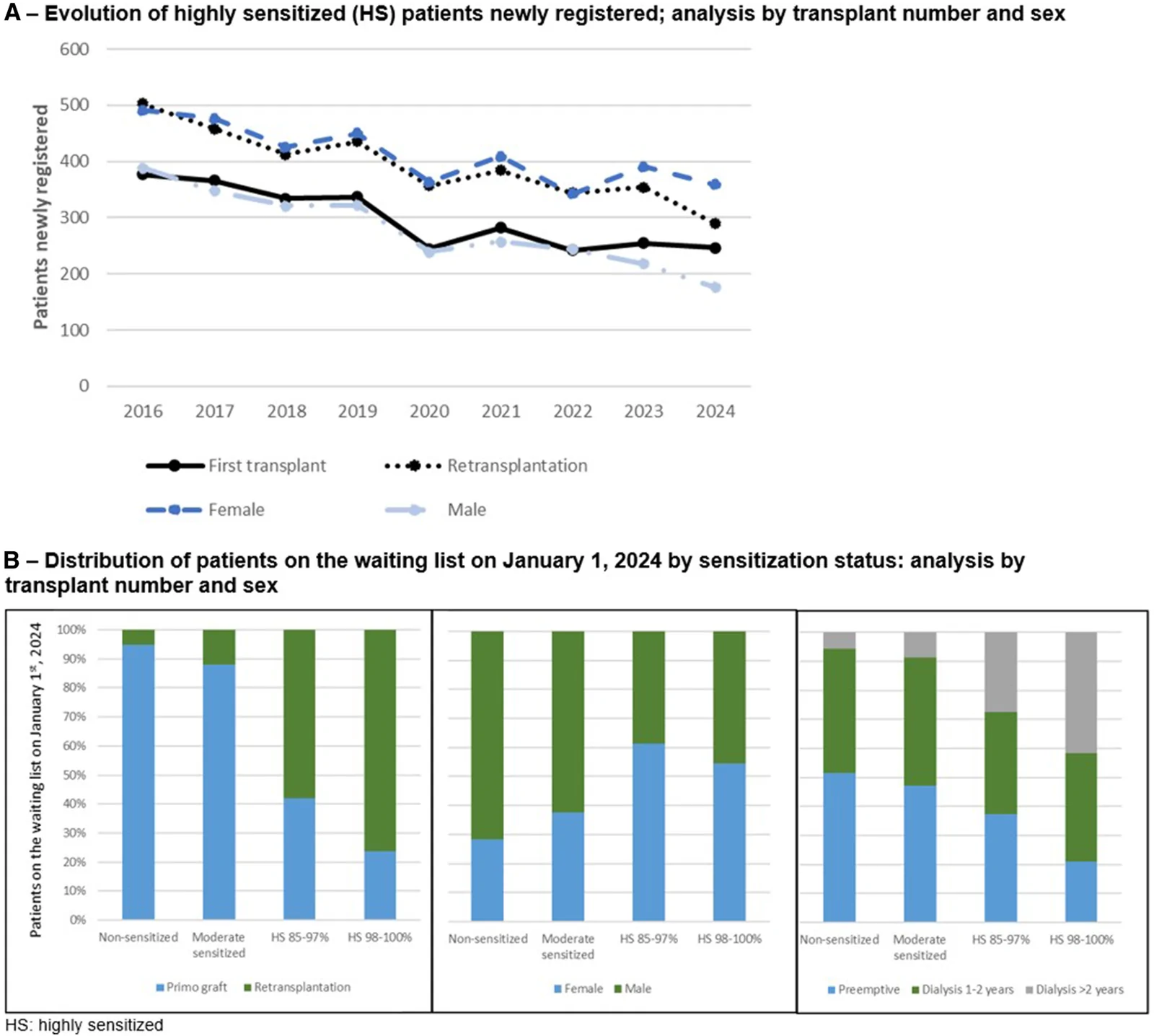
Characteristics of patients by sensitization. **(A)** Evolution of highly sensitized (HS) patients newly registered; analysis by transplant number and sex. **(B)** Distribution of patients on the waiting list on January 1, 2024 by sensitization status: analysis by transplant number and sex.

From 2016 to 2025, the number of HS patients active on the kidney waiting list decreased from 2,024 to 1,866 (from 28% to 17%, Figure 1; Supplementary Table S1; MS recipients in Supplementary Table S2; reasons for delisting in Supplementary Table S3). Annual new registrations also decreased steadily, representing a decline from 17% to 9%. These decreases were observed without any modification to the method used to calculate cRF. The total number of transplants ranged from 331 to 560 annually, with a marked decline after 2019. Transplants were mainly from DBDD; living donor and controlled donation after circulatory determination of death represented a small but stable minority throughout the period. When restricting the analysis to new registration candidates to avoid the effect of long-waiting patients with very high cRF, we found a reduction in the number of eligible HS candidates as first-transplant (−35%) and retransplant patients (−43%) (Figure 2A). This decrease in proportion was more pronounced for male than female candidates (−55% vs. −27%).

Among prevalent patients actively listed on January 1 of each year, the clinical characteristics differed significantly between HS and non-sensitized candidates. The distribution of sensitization levels varied markedly across recipient subgroups (Figure 2B; Supplementary Figure S2). Non-sensitized recipients predominated among first transplant candidates and preemptive recipients, whereas HS patients (HS 98%–100%) were overrepresented among retransplantation and long-term dialysis (>2 years) groups. Moderate sensitization (MS) and intermediate highly sensitization (cRF 85%–97%) accounted for substantial proportions in female and male recipients as well as those with 1–2 years of dialysis. Overall, retransplantation and prolonged dialysis were associated with an increased burden of sensitization as compared with first transplants or shorter dialysis exposure. Accordingly, retransplantation accounted for 69% of listings among HS candidates versus only 12% among non-sensitized candidates. Women represented 57% of HS candidates versus 34% of non-sensitized candidates. Furthermore, 36% of HS patients had been on dialysis for >2 years versus 21% of non-sensitized candidates.

Longitudinal analyses of new registrations are in Supplementary Figures S3–S5.

### Access to kidney transplantation

**FIGURE 3:**
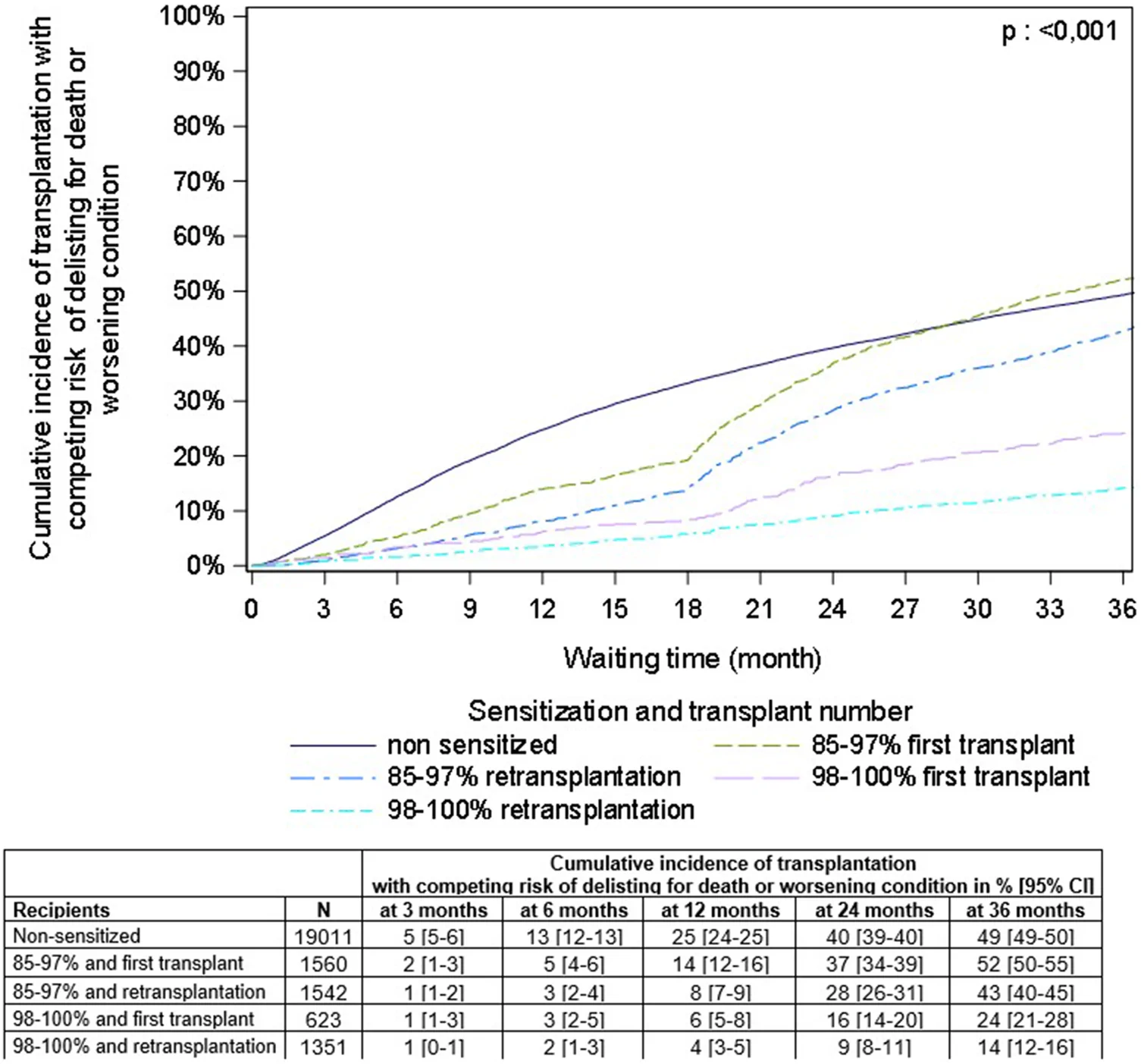
Cumulative incidence of access to transplantation by sensitization status and transplant number.

The cumulative incidence of kidney transplantation varied markedly by cRF level and transplant number (p < 0.001) (Figure 3). Non-sensitized recipients showed the highest access to transplantation: 49% [49–50] at 36 months. Among HS recipients, those with peak cRF 85%–97% undergoing a first transplant had comparable access: 52% [50–55] transplanted at 36 months. In contrast, retransplant candidates with peak cRF 85%–97% had lower access at 43% [40–45]. Outcomes were poorest for patients with peak cRF 98%–100%: 24% [21–28] for first transplants and only 14% [12–16] for retransplants at 36 months. Overall, access decreased with higher cRF and was consistently lower with retransplantation versus first transplantation. We note a clear inflection in the access curve at about 18 months after waitlist registration, corresponding to the time when patients become eligible for the national AMP. Notably, despite substantial differences in access to transplantation, the cumulative incidence of delisting for death or worsening condition remained low across sensitization categories at 36 months, from 8% [7–10] to 10% [8–13] frequencies. The cumulative incidence of delisting for death or worsening condition is detailed in Supplementary Figure S6. The cumulative incidence of kidney transplantation for MS group A and B is detailed in Supplementary Figure S7.

### Post-transplant survival analysis

From 2016 to 2022, 2,985 single-organ kidney transplantations were performed in adult HS recipients from DBDDs in France (Supplementary Figure S8).

Known risk factors for graft failure were not equally distributed between non-sensitized and HS recipients or between AMP and non-AMP recipients (Supplementary Table S4).

As compared with non-sensitized recipients, HS recipients were more frequently younger, female, undergoing retransplantation, and on dialysis; they more frequently had blood group B and a longer dialysis duration. They less often had a body mass index >30 kg/m^2^, comorbidities, and a diabetic cause for end-stage kidney disease. They more often received right-side kidneys, received kidneys from donors who were 45–69 years old, had died from vascular causes, and had blood group O, and more often had longer cold ischemia times than non-sensitized recipients.

As compared with HS non-AMP recipients, HS AMP recipients were older and had more comorbidities. They were less often pre-emptive but had shorter dialysis duration. HS AMP recipients more often received kidneys from older donors and had longer cold ischemia times. Non-AMP recipients more often had previous history of kidney transplants, more frequently a peak cRF of 99%–100% (26% vs. 14%), and transplantations with more HLA mismatches.

**FIGURE 4:**
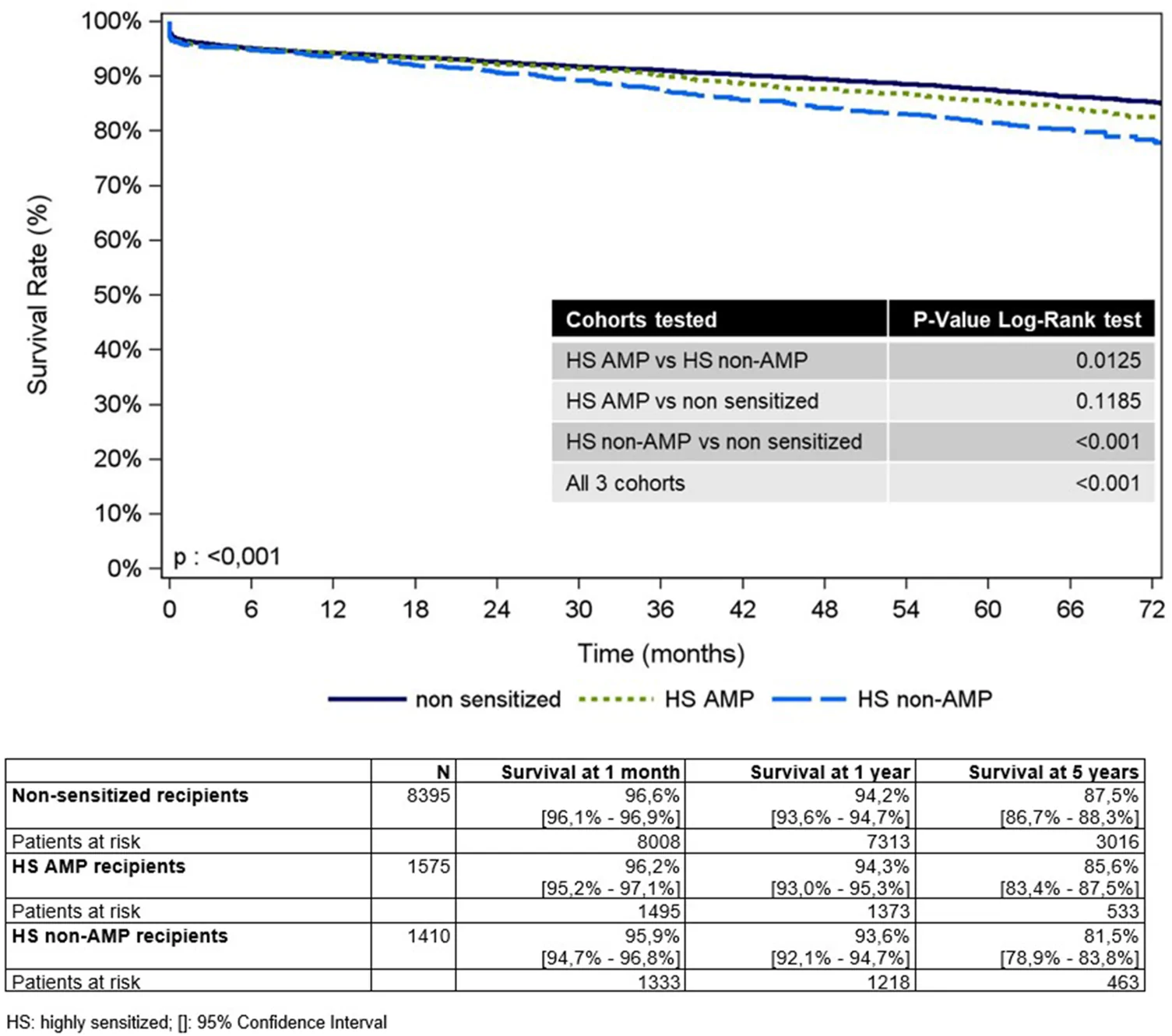
Kidney graft survival with death censored in highly sensitized (HS) Acceptable Mismatch Program (AMP) recipients and HS non-AMP recipients vs. non-sensitized recipients (observation end date: January 1, 2025, Kaplan-Meier method).

Overall survival death censored rates were comparable for HS AMP and non-sensitized recipients during follow-up, with no significant difference on log-rank testing (p = 0.1185) (Figure 4). In contrast, survival was significantly lower among HS non-AMP than HS AMP recipients (p = 0.0125). Kidney graft survival with death censored for MS groups A and B is detailed in Supplementary Figure S9. Patient survival by sensitization is detailed in Supplementary Figure S10.

**FIGURE 5:**
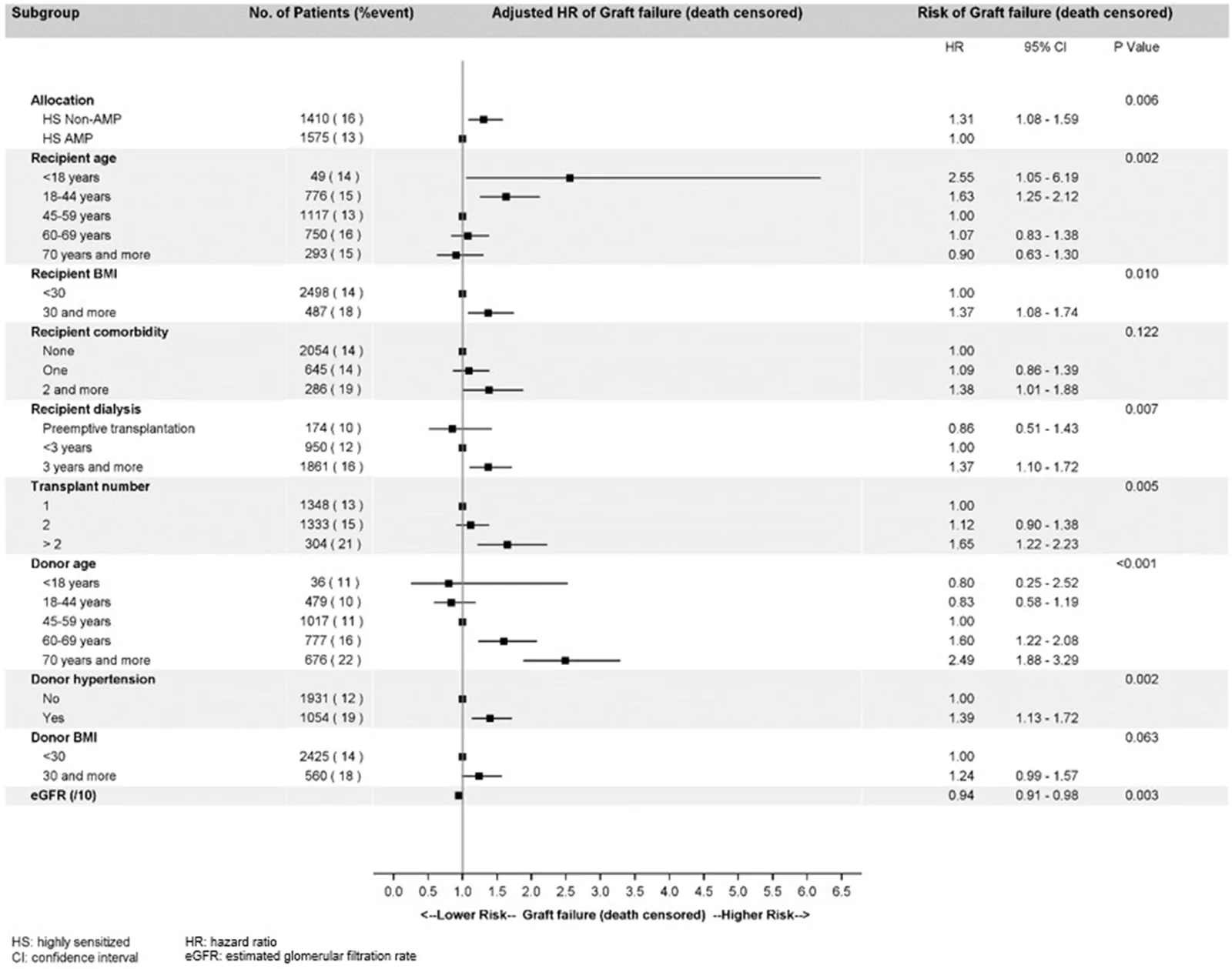
Forest plot of risk factors of kidney graft survival in the highly sensitized recipient cohort with death censored (Cox model, observation end date: January 1, 2025).

After adjustment for donor and recipient characteristics with a Cox model, risk of graft failure was associated with HS non-AMP allocations (HR 1.19 [95% CI 1.04–1.36]; 1.31 (95% CI 1.08–1.59) with death censored; Supplementary Figure 11 and Figure 5). Three sensitivity analysis were performed. First, a complete-case analysis excluded transplants with missing data for 1,398 HS non-AMP and 1,553 HS AMP recipients. The risk of graft failure was associated with HS non-AMP allocations (HR 1.19 [95% CI 1.04–1.36]; 1.30 [95% CI 1.07–1.58] with death censored). Second, we added a center effect. Again, the risk of graft failure was associated with HS non-AMP allocations (HR 1.17 (95% CI 1.02–1.34]; 1.28 [95% CI 1.05–1.55] with death censored). Finally, in an analysis restricted to right-side donor donation, the risk of graft failure was associated with HS non-AMP allocations (HR 1.20 [95% CI 1.01–1.42]; 1.31 [95% CI 1.04–1.65] with death censored). Other independent predictors of death-censored graft loss included young recipient age, high body mass index, multiple comorbidities, dialysis ≥3 years, transplant number ≥2, older donor age, donor hypertension, and low donor eGFR.

The kidney graft survival with death censored for HS AMP, HS non-AMP without DSA, and HS non-AMP with DSA recipients is detailed in Supplementary Figure S12. Acute or chronic rejection frequency for HS AMP, HS non-AMP without DSA, and HS non-AMP with DSA recipients is detailed in Supplementary Table S4.

## Discussion

This study demonstrates that improved death-censored graft survival in HS patients is achieved with the French AMP, offering post-transplant survival comparable to that for non-HS recipients transplanted under the standard allocation scheme. Despite an increased risk of rejection, HS recipients transplanted under the AMP achieved significantly better 5-year graft survival than HS patients transplanted outside the AMP, without additional desensitization strategies. At comparable cRF levels, HS AMP candidates awaiting a first transplant had better access to transplantation than those awaiting retransplantation, which reflects higher class-II HLA immunization and fewer permissible class-II HLA Ag in the latter group. The profile of patients who benefited most from access programs included candidates with common HLA phenotypes with cRF >97%, first-transplant recipients, patients with lower class-II HLA sensitization, particularly against HLA-DQβ Ag, and those with a greater number of permissible mismatches in class-II HLA, including DR and DQβ. In contrast, HS non-AMP patients more frequently had a history of prior kidney transplantation. This observation reflects listing for retransplantation commonly being associated with increased class-II HLA immunization and DSAs. In this context, selected patients might be under an immunologic forgiveness policy to facilitate access to a subsequent graft.

Over the past 10 years, the proportion of HS patients on the active waiting list decreased markedly, from 29% to 17%. This improvement is mainly the result of two combined factors.

The first factor is better HLA matching strategies, especially the emphasis on high-quality class-II matching, which is included in our French National Kidney Allocation Score and is strongly weighted for younger recipients. This strategy, implemented since the nationalization of the kidney allocation score [3], helps reduce the creation of hyperimmunized patients after a previous graft failure by introducing immunologic sparing for the youngest recipients. HLA compatibility, particularly class-II HLA, carries a high coefficient in the score, especially for the youngest patients; the weight then decreases with recipient age. By promoting HLA class-II matching among young adult recipients, the number of hyperimmunized re-registrants has been limited and post-transplant sensitization reduced.

The second factor in the reduced proportion of HS patients on the active waiting list is improved allelic resolution of anti-HLA antibody testing and donor HLA typing and, a better interpretation based on SFHI and European Federation for Immunogenetics recommendations [8]. Recent recommendations have helped refine HLA antibody characterization by encouraging high-resolution, allele-level analysis while maintaining a critical approach to the risk of false-positive SAFB results, particularly via the evaluation of autoreactivity and assay artefacts [9–14]. These guidelines also emphasize avoiding the blanket exclusion of all DQβ Ag carrying a given DQα epitope and instead recommend focusing on the corresponding frequent (haplotype-based) DR-associated specificities in the presence of clinically relevant anti-DQα reactivity. Additional measures, such as improved technical standardization across laboratories, reduction of inter-assay variability, and the use of epitope-based interpretation tools, have been implemented, thereby enhancing the accuracy of unacceptable Ag assignment [12].

Collectively, these advances have substantially reduced the number of listed unacceptable HLA Ag and, consequently, the proportion of candidates classified as HS. These developments could be effectively implemented with the national adoption of the pretransplant virtual crossmatch, which serves as the final and essential safety check before accepting a graft. This implementation was facilitated by the integration, within the CRISTAL donor-management platform, of allele-level donor HLA typing together with national guidelines defining interpretation criteria and technical warning thresholds [6].

The French experience can be viewed as a succession of major immunologic milestones: 1) nationwide implementation of SAFB-based unacceptable Ag in 2009; 2) introduction of a cRF directly derived from SAFB-defined unacceptable Ag in late 2009; 3) incorporation of anti-HLA-DQ antibodies into kidney allocation and cRF calculation in 2009; 4) introduction of the new French Kidney Allocation System in 2015 with improved HLA matching, particularly for younger recipients; 5) nationwide implementation of virtual crossmatch and intermediate/high-resolution HLA typing from 2016 onward; and 6) progressive refinement of antibody interpretation by the exclusion of denatured HLA antibodies and isolated allele-specific sensitizations. Together, these successive developments likely explain why France experienced an early increase in the proportion of HS candidates, followed by a sustained decline over the last few years. Future changes in national consensus regarding the integration of anti-HLA-Cw and -DP antibodies into cRF calculation and the allocation algorithm for HS patients may again modify the epidemiology of HLA sensitization, particularly among retransplant candidates.

Although the cRF remains the cornerstone indicator for defining immunologic sensitization and access to national priority, it does not fully capture the structural difficulty of identifying a compatible donor for a given candidate. In this respect, complementary indicators such as the PMD, which estimates the number of compatible and well-matched donors among actually utilized deceased donors in recent years, provide additional insight into real-world transplantability. Patients with similar cRF values may exhibit markedly different PMD profiles, reflecting differences in HLA phenotype frequency and compatibility constraints. This distinction is particularly relevant for candidates with rare HLA phenotypes or limited acceptable mismatches, who may remain disadvantaged despite comparable cRF levels.

HS patients with a cRF >85% but <98% have good access to kidney transplantation. Their transplant rate increases markedly after 18 months on the waiting list and eventually reaches that of non-immunized first transplant candidates. Among candidates with cRF 98%–100%, most are awaiting retransplantation. Of the quarter of candidates who are first-transplant patients, most are women sensitized (90%) through pregnancy and/or transfusion, with broad class-I HLA immunization. In this group, first-time registrants have higher transplant rates, reaching 24% by 3 years. Overall, patients with common HLA phenotypes, particularly first-transplant candidates, including women, benefit most, because they have fewer unacceptable class-II Ag and more acceptable mismatches, especially regarding HLA-DQβ Ag. Finally, the AMP program also addresses sex inequities, because women are more frequently sensitized than men.

After adjustment, risk of death-censured graft failure was 31% higher for HS non-AMP than HS AMP recipients. Other significant risk factors included young recipient age, high body mass index, multiple comorbidities, prolonged dialysis duration (>3 years), retransplantation, and older donors with hypertension. Young recipient age has consistently emerged as a risk factor in numerous studies, because immune responsiveness tends to be more vigorous in younger individuals and this group also has a higher likelihood of non-adherence. Cold ischemia time also plays an important role, as it does in all kidney transplants, by inducing damage-associated molecular patterns via ischemia–reperfusion injury, leading to the overexpression of HLA Ag on endothelial cells. Moreover, recent mechanistic reviews highlight that ischemia–reperfusion injury triggers endothelial activation and complex innate–adaptive immune interactions that contribute to both acute and antibody-mediated rejection in kidney transplantation [15].

The French allocation framework has historically embedded the principle that priority for HS candidates should be granted only when the proposed transplant is expected to carry an acceptable immunologic risk and outcomes comparable to those observed under standard allocation rules. Therefore, national priority has never been conceived as an unconditional advantage but rather as a mechanism to restore equity while preserving optimal graft utility. In this context, the AMP fits precisely within this regulatory philosophy because it is able to expand donor options: it allows for improved access for difficult-to-match candidates without increasing rejection risk or compromising long-term graft survival. Several notable differences distinguish the French AMP from the Eurotransplant AMP. Both rely on a cRF threshold of 85% based on real donor data, although the Eurotransplant AMP is accessible after 2 years of waiting time on dialysis and centralizes eligibility assessment within a single reference laboratory in Leiden, where the immunologic profile is determined not just from SAFB testing but also includes the HLA Matchmaker [1, 16, 17]. This algorithm identifies acceptable Ag predicted to carry a very low risk of *de novo* DSAs formation, based on shared or non-permissive eplets, whereas the French AMP prioritizes identifying donors without any historical or current DSA on the day of transplantation. Despite these methodological differences, outcomes remain excellent in both programs, as shown in the most recent Eurotransplant reports [11, 18, 19]. A final distinction is that the French AMP does not incorporate anti-HLA-Cw or -DP antibodies in its exclusion criteria because of the lack of national consensus on their interpretation, particularly given the high proportion of false-positive anti-HLA-Cw results in first kidney transplant candidates [10, 20]. These antibodies are instead managed at the time of organ offer via pretransplant virtual crossmatch assessment and, when necessary, confirmatory cellular testing.

Nevertheless, a subset of candidates remains poorly served by current allocation strategies, including those with extremely high cRF values combined with rare HLA phenotypes, absence of common linkage-disequilibrium haplotypes, or homozygosity at multiple HLA loci. For these patients, even acceptable mismatch–based approaches may fail to generate compatible offers. These limitations underscore the need to shift the focus upstream, by preventing the emergence of such extreme immunologic profiles via improved HLA matching, particularly at the class-II level, beginning at the time of the first transplant for the youngest recipients. In the future, the number of such patients could be reduced if a minimum level of HLA class-II compatibility, or the inclusion of less immunogenic class-II matching, is systematically implemented in standard allocation systems. Another elegant approach would be to preferentially allocate both kidneys from a single donor with homozygous HLA class-II typing to HS candidates with cRF 99%–100% who have been on dialysis for several years. From the French CRISTAL Registry, we found 193 DBDDs aged 18–65 years who matched this hypothesis from 2021 to 2023. An analysis of the fate of these “golden” grafts showed that a significant proportion are often transplanted locally to less sensitized recipients with few or no years on dialysis, even though, because of the homozygous HLA DR-DQβ haplotype, the graft may represent the only compatible organ for HS patients waiting for >5 years.

Recent recommendations have also proposed strategies for these long-waiting patients without an immediately compatible donor, including immunologic forgiveness policies (Ag delisting after antibody decline or disappearance over time or antibodies that do not activate complement) [21, 22] or carefully monitored desensitization protocols [23, 24], based on simulations from deceased donor databases. Indeed, HS non-AMP recipients are at increased immunologic risk and require more intensive immunosuppressive regimens, with associated infectious or oncologic complications, yet outcomes remain acceptable, providing a viable option when no alternative is available.

A key limitation of this study is its observational design, which precludes definitive causal inference and leaves the possibility of residual confounding despite adjustment for measured covariates. In addition, mechanistic interpretations should be approached with caution because the present analysis could not reliably assess rejection episodes, DSAs, *de novo* DSAs, or immunologic causes of graft loss comprehensively. The CRISTAL registry, managed by the *Agence de la biomédecine*, offers nationwide coverage of organ allocation and transplantation activity in France but is primarily designed to support allocation processes and long-term outcome monitoring rather than detailed clinical research. As a result, although the registry includes fields for acute and chronic rejection, antibody-mediated rejection, and cellular rejection, with or without DSAs, completion of these fields is not mandatory and the data quality is not subject to the same level of control as allocation-related data. Likewise, the specific cause of graft loss is incompletely recorded. Consequently, the reporting of immunologic complications is not exhaustive, which limits the robustness of any mechanistic analysis between preformed sensitization and graft outcomes. To further explore this issue, we performed an additional analysis by the presence of historical preformed DSAs that had been subsequently removed from the list of unacceptable antigens during the waiting period. This analysis showed a significant difference in death-censored graft survival between HS AMP recipients, HS non-AMP recipients without such historical DSA, and HS non-AMP recipients with historical DSAs, with 5-year death-censored graft survival rates of 85.6% [83.4–87.5], 81.7% [79.0–84.0], and 75.4% [61.2–85.0], respectively (p = 0.024). In addition, among graft losses for which rejection was reported as acute or chronic, the corresponding proportions were 30%, 38%, and 43%, although these data should be interpreted with caution given incomplete reporting. Taken together, these findings are consistent with a possible role of immunologic memory and preformed DSAs, but they do not allow firm mechanistic conclusions.

In conclusion, the French AMP represents a major advancement in kidney transplantation for HS patients, substantially improving their access to suitable donors while maintaining post-transplant outcomes comparable to those of non-sensitized recipients. More broadly, our findings illustrate that acceptable mismatch–based allocation represents an effective compromise between equity and utility. By selectively expanding donor compatibility while maintaining stringent immunologic safety criteria, the AMP restores access to transplantation for HS candidates without compromising graft outcomes. This balance is particularly critical in systems in which organ scarcity mandates that increased equity for disadvantaged patients must not come at the expense of predictable graft failure. By facilitating transplantation for women, who are disproportionately represented among HS candidates due to prior pregnancies, the program also contributes to reducing sex-related inequities in organ allocation [25]. More broadly, these results underscore that the most effective long-term strategy remains careful immunologic management at the time of transplantation, particularly through high-quality HLA class-II matching in young recipients, which minimizes alloimmune risk and maximizes graft longevity. Overall, the AMP exemplifies how tailored allocation policies, informed by precise immunologic profiling, can optimize both equity and clinical outcomes in transplantation.

## Data Availability

All data used in this study were extracted from the CRISTAL registry, coordinated and supported by the French Agence de la biomédecine. External access for research to national data is regulated by a scientific committee of the French Agence de la biomédecine, which analyses each request. Thus, data cannot be made publicly available due to legal restrictions. Data are available upon reasonable request. If readers need information about the data from the CRISTAL registry, they can contact Nicolas Chatauret (nicolas.chatauret@biomedecine.fr).

## Footnotes

1 Virtual Panel-Reactive Antibodies is based on the frequency of unacceptable antigens in the theoretical donor population.

2 cRF estimates the proportion of HLA-incompatible deceased donors among all transplanted kidney deceased donors (or actual deceased donors within the same blood group, over a specific period).
