# Supplementary material for: Improved access to kidney transplantation with favorable graft survival in highly sensitized candidates under the French acceptable mismatch program
Source: Transpl Int. 2026 Sep 8;39:16762. doi: 10.3389/ti.2026.16762 (PMC13593581; doi:10.3389/ti.2026.16762)
Supplement: Supplementary file 1 [file Supplementaryfile1.docx]

**Supplementary Tables and Figures**

**Supplementary Figure 1. Directed acyclic graph showing assumed associations between highly sensitized (HS) allocation, survival graft, baseline donor or recipient characteristics, and transplant conditions**

Because of the acceptable mismatch program (AMP) and different epidemiology depending on recipient selection, confounders and mediators exist between the exposure variable (HS allocation) and the outcome (survival graft).

*HS: highly sensitized; AMP: acceptable mismatch program; HLA: human leukocyte antigen; DSA: donor-specific antibodies*

**Supplementary Table 1. Waiting list and transplant activity of highly sensitized (HS) patients by year of study**

|  | **2016** | **2017** | **2018** | **2019** | **2020** | **2021** | **2022** | **2023** | **2024** | **2025** |
| --- | --- | --- | --- | --- | --- | --- | --- | --- | --- | --- |
| **Frequency of HS patients** | | | | | | | | | | |
| Active on the waiting list on January 1 | 2024 | 2181 | 2164 | 2114 | 2071 | 2051 | 2059 | 1985 | 1923 | 1866 |
| Inactive on the waiting list on January 1 | 1262 | 1307 | 1426 | 1467 | 1472 | 1492 | 1522 | 1531 | 1511 | 1424 |
| New registration on the waiting list | 880 | 824 | 746 | 772 | 602 | 666 | 586 | 609 | 535 |  |
| Died on the waiting list | 102 | 104 | 113 | 137 | 150 | 127 | 129 | 109 | 107 |  |
| Delisting from the waiting list (all causes) | 83 | 90 | 115 | 113 | 121 | 91 | 101 | 155 | 130 |  |
| *Including delisting for worsening condition* | *53* | *56* | *71* | *62* | *61* | *48* | *53* | *100* | *70* |  |
| Total transplants | 493 | 528 | 527 | 560 | 331 | 410 | 421 | 427 | 442 |  |
| Living donor transplants | 55 | 59 | 45 | 41 | 18 | 27 | 20 | 22 | 26 |  |
| DBDD transplants | 435 | 468 | 477 | 511 | 309 | 376 | 394 | 401 | 405 |  |
| cDCDD transplants | 3 | 1 | 5 | 7 | 4 | 7 | 7 | 4 | 11 |  |
| **Percentage of HS patients** | | | | | | | | | | |
| Active on the waiting list on January 1 | 28% | 28% | 28% | 26% | 24% | 23% | 21% | 20% | 18% | 17% |
| Inactive on the waiting list on January 1 | 24% | 22% | 22% | 20% | 19% | 18% | 18% | 17% | 16% | 14% |
| New registration on the waiting list | 17% | 15% | 14% | 14% | 12% | 12% | 11% | 10% | 9% |  |
| Died on the waiting list | 29% | 25% | 23% | 26% | 23% | 18% | 17% | 16% | 17% |  |
| Delisting from the waiting list (all causes) | 24% | 25% | 25% | 21% | 23% | 17% | 17% | 18% | 16% |  |
| *Including delisting for worsening condition* | 25% | 27% | 25% | 19% | 19% | 16% | 16% | 19% | 15% |  |
| Total transplants | 14% | 14% | 15% | 15% | 13% | 13% | 12% | 12% | 12% |  |
| Living donor transplants | 10% | 10% | 8% | 8% | 5% | 5% | 4% | 4% | 4% |  |
| DBDD transplants | 15% | 16% | 17% | 18% | 16% | 16% | 16% | 16% | 15% |  |
| cDCDD transplants | 3% | 1% | 2% | 2% | 2% | 2% | 2% | 1% | 2% |  |

*HS: highly sensitized; DBDD: donation after brain determination of death; cDCDD: controlled donation after circulatory determination of death*

**Supplementary Table 2. Waiting list and transplant activity of moderately sensitized (MS) patients by year of study**

|  | **2016** | **2017** | **2018** | **2019** | **2020** | **2021** | **2022** | **2023** | **2024** | **2025** |
| --- | --- | --- | --- | --- | --- | --- | --- | --- | --- | --- |
| **MS group A** |  |  |  |  |  |  |  |  |  |  |
| **Frequency of “MS group A” patients** |  |  |  |  |  |  |  |  |  |  |
| Active on the waiting list on January 1st | 2473 | 2541 | 2579 | 2657 | 2870 | 3055 | 3398 | 3523 | 3819 | 3965 |
| Inactive on the waiting list on January 1st | 1813 | 2031 | 2299 | 2548 | 2694 | 3012 | 3095 | 3292 | 3458 | 3575 |
| New registration on the waiting list | 1731 | 1892 | 1909 | 1950 | 1728 | 1978 | 1985 | 2189 | 2043 |  |
| Died on the waiting list | 107 | 141 | 156 | 167 | 214 | 244 | 258 | 221 | 233 |  |
| Delisting from the waiting list (all causes) | 104 | 113 | 157 | 187 | 177 | 195 | 206 | 275 | 251 |  |
| *Including delisting for worsening condition* | *64* | *67* | *105* | *124* | *114* | *113* | *109* | *158* | *153* |  |
| Total transplants | 1234 | 1332 | 1269 | 1237 | 834 | 1113 | 1199 | 1231 | 1296 |  |
| living donor transplants | 234 | 225 | 193 | 193 | 138 | 164 | 174 | 186 | 211 |  |
| DBD transplants | 955 | 1038 | 990 | 911 | 615 | 807 | 876 | 868 | 887 |  |
| cDCD transplants | 40 | 66 | 82 | 129 | 81 | 142 | 149 | 177 | 198 |  |
| **Percentage of “MS group A” patients** |  |  |  |  |  |  |  |  |  |  |
| Active on the waiting list on January 1st | 34% | 33% | 33% | 33% | 33% | 34% | 35% | 36% | 36% | 36% |
| Inactive on the waiting list on January 1st | 35% | 35% | 35% | 35% | 35% | 36% | 36% | 36% | 36% | 36% |
| New registration on the waiting list | 33% | 35% | 35% | 35% | 36% | 36% | 37% | 36% | 35% |  |
| Died on the waiting list | 30% | 34% | 32% | 32% | 33% | 34% | 34% | 33% | 37% |  |
| Delisting from the waiting list (all causes) | 30% | 31% | 34% | 35% | 34% | 36% | 35% | 32% | 31% |  |
| *Including delisting for worsening condition* | 30% | 32% | 36% | 39% | 36% | 37% | 32% | 30% | 32% |  |
| Total transplants | 34% | 35% | 36% | 34% | 32% | 34% | 36% | 35% | 34% |  |
| Living donor transplants | 41% | 37% | 36% | 38% | 35% | 33% | 34% | 33% | 35% |  |
| DBD transplants | 33% | 35% | 36% | 33% | 32% | 34% | 36% | 35% | 34% |  |
| cDCD transplants | 47% | 37% | 38% | 40% | 32% | 37% | 37% | 38% | 37% |  |
|  | 2016 | 2017 | 2018 | 2019 | 2020 | 2021 | 2022 | 2023 | 2024 | 2025 |
| **MS group B** |  |  |  |  |  |  |  |  |  |  |
| **Frequency of “MS group B” patients** |  |  |  |  |  |  |  |  |  |  |
| Active on the waiting list on January 1st | 157 | 177 | 209 | 211 | 195 | 135 | 136 | 123 | 103 | 84 |
| Inactive on the waiting list on January 1st | 87 | 109 | 113 | 109 | 102 | 93 | 91 | 83 | 81 | 57 |
| New registration on the waiting list | 62 | 53 | 48 | 63 | 25 | 26 | 15 | 19 | 9 |  |
| Died on the waiting list | 2 | 1 | 6 | 8 | 13 | 7 | 3 | 4 | 5 |  |
| Delisting from the waiting list (all causes) |  | 2 | 3 | 3 | 9 | 2 | 5 | 4 | 8 |  |
| Including delisting for worsening condition |  | 1 |  |  | 5 |  | 2 | 3 | 6 |  |
| Total transplants | 18 | 14 | 41 | 75 | 72 | 18 | 28 | 33 | 39 |  |
| Living donor transplants | 1 | 1 | 8 | 9 | 8 | 2 | 5 | 1 | 3 |  |
| DBD transplants | 17 | 12 | 33 | 62 | 60 | 16 | 21 | 30 | 33 |  |
| cDCD transplants |  | 1 |  | 3 | 4 |  | 2 | 2 | 3 |  |
| **Percentage of “MS group B” patients** |  |  |  |  |  |  |  |  |  |  |
| Active on the waiting list on January 1st | 2% | 2% | 3% | 3% | 2% | 2% | 1% | 1% | 1% | 1% |
| Inactive on the waiting list on January 1st | 2% | 2% | 2% | 2% | 1% | 1% | 1% | 1% | 1% | 1% |
| New registration on the waiting list | 1% | 1% | 1% | 1% | 1% | 0% | 0% | 0% | 0% |  |
| Died on the waiting list | 1% | 0% | 1% | 2% | 2% | 1% | 0% | 1% | 1% |  |
| Delisting from the waiting list (all causes) | 0% | 1% | 1% | 1% | 2% | 0% | 1% | 0% | 1% |  |
| Including delisting for worsening condition | 0% | 0% | 0% | 0% | 2% | 0% | 1% | 1% | 1% |  |
| Total transplants | 0% | 0% | 1% | 2% | 3% | 1% | 1% | 1% | 1% |  |
| Living donor transplants | 0% | 0% | 1% | 2% | 2% | 0% | 1% | 0% | 1% |  |
| DBD transplants | 1% | 0% | 1% | 2% | 3% | 1% | 1% | 1% | 1% |  |
| cDCD transplants | 0% | 1% | 0% | 1% | 2% | 0% | 0% | 0% | 1% |  |

*MS group A: moderately sensitized patients with peak cRF <85% and current cRF <85%*

*MS group B: moderately sensitized patients with peak cRF ≥85% and current cRF ≤70%
DBDD: donation after brain determination of death*

*cDCDD: controlled donation after circulatory determination of death*

**Supplementary Figure 2. Distribution of patients on the waiting list on January 1, 2024 by sensitization status: analysis by transplant number and sex**

*HS: highly sensitized*

**Supplementary Figure 3. Evolution patients newly registered according to transplant number**

**Supplementary Figure 4. Evolution patients newly registered according to cRF in retransplant candidates**

**Supplementary Figure 5. Evolution patients newly registered according to age in retransplant candidates**

**Supplementary Table 3. Reason of delisting other than death between 2016 and 2024 of highly sensitized patients**

| **Reason of delisting** | **Frequency** | **Percent** |
| --- | --- | --- |
| Worsening condition other than the initial indication* | 446 | 44.6% |
| Worsening condition of the initial indication* | 128 | 12.8% |
| Improvement of the initial indication | 19 | 1.9% |
| Registration cancelled | 7 | 0.7% |
| Patient's personal decision | 388 | 38.8% |
| Transplanted abroad | 11 | 1.1% |

**Reasons corresponding to delisting for worsening condition*

**Supplementary Figure 6. Cumulative incidence** **of delisting for death or worsening condition according to sensitization and transplant number**

|  | | **Cumulative incidence of delisting for death or worsening condition  with competing risk of transplantation in % [95% CI]** | | | | |  |
| --- | --- | --- | --- | --- | --- | --- | --- |
|  |  |  |  |  |  |  |  |
| **Recipients** | **N** | **at 3 months** | **at 6 months** | **at 12 months** | **at 24 months** | **at 36 months** |  |
| non sensitized | 19011 | 1 [1-1] | 2 [1-2] | 3 [3-3] | 6 [6-7] | 9 [9-10] |  |
| 85-97% and first transplant | 1560 | 0 [0-1] | 1 [1-2] | 3 [2-4] | 6 [5-7] | 9 [8-11] |  |
| 85-97% and retransplantation | 1542 | 0 [0-1] | 1 [0-1] | 2 [1-2] | 5 [4-6] | 8 [7-10] |  |
| 98-100% and first transplant | 623 | 0 [0-1] | 1 [1-3] | 3 [2-4] | 7 [5-9] | 10 [8-13] |  |
| 98-100% and retransplantation | 1351 | 0 [0-1] | 1 [0-1] | 2 [1-3] | 5 [4-6] | 9 [7-10] |  |

**Supplementary Figure 7. Cumulative incidence of access to transplants according to sensitization**

|  | | **Cumulative incidence of transplantation  with competing risk of delisting for death or worsening condition in % [95% CI]** | | | | |  |
| --- | --- | --- | --- | --- | --- | --- | --- |
|  |  |  |  |  |  |  |  |
| **Recipients** | **N** | **at 3 months** | **at 6 months** | **at 12 months** | **at 24 months** | **at 36 months** |  |
| non sensitized | 19011 | 5 [5-6] | 13 [12-13] | 25 [24-25] | 40 [39-40] | 49 [49-50] |  |
| MS group A | 13173 | 3 [3-4] | 8 [7-8] | 17 [17-18] | 31 [30-32] | 42 [41-42] |  |
| MS group B | 292 | 0 [0-2] | 2 [1-4] | 8 [5-11] | 22 [17-27] | 33 [28-38] |  |
| HS | 5076 | 1 [1-2] | 3 [3-4] | 8 [8-9] | 24 [23-26] | 36 [34-37] |  |

HS: highly sensitized patients with peak cRF ≤85% and current cRF ≤70%

MS group a: moderately sensitized patients with peak cRF <85% and current cRF <85%

MS group b: moderately sensitized patients with peak cRF ≥85% and current cRF ≤70%

**Supplementary Figure 8. Enrollment and outcomes of survival analysis of non sensitized and highly sensitized (HS) recipients**

*DBDD: donation after brain determination of death*

*HS: highly sensitized*

*AMP: acceptable mismatch program*

**Supplementary Table 4. Comparison of highly sensitized (HS) recipients versus non-sensitized recipients and HS acceptable mismatch program (AMP) versus HS non-AMP recipients**

|  |  | **Non-sensitized vs HS** | | | **HS AMP vs HS non-AMP** | | | |
| --- | --- | --- | --- | --- | --- | --- | --- | --- |
|  |  | **Non-sensitized** | **HS** | **P-value** | **HS AMP*** | **HS Non-AMP*** | **P-value** | **Standardized difference** |
| Recipient age, years | <18 | 289 (3.4) | 49 (1.6) | <0.01 | 28 (1.8) | 21 (1.5) | <0.01 | 0.1717 |
|  | 18-44 | 1888 (22.5) | 776 (26) |  | 391 (24.8) | 385 (27.3) |  |  |
|  | 45-59 | 2433 (29) | 1117 (37.4) |  | 550 (34.9) | 567 (40.2) |  |  |
|  | 60-69 | 1974 (23.5) | 750 (25.1) |  | 446 (28.3) | 304 (21.6) |  |  |
|  | ≥ 70 | 1811 (21.6) | 293 (9.8) |  | 160 (10.2) | 133 (9.4) |  |  |
| Sex | Male | 5984 (71.3) | 1254 (42) | <0.01 | 686 (43.6) | 568 (40.3) | 0.07 | 0.0663 |
|  | Female | 2411 (28.7) | 1731 (58) |  | 889 (56.4) | 842 (59.7) |  |  |
| Recipient blood group | A | 3861 (46) | 1267 (42.4) | <0.01 | 588 (37.3) | 679 (48.2) | <0.01 | 0.2599 |
|  | AB | 397 (4.7) | 152 (5.1) |  | 89 (5.7) | 63 (4.5) |  |  |
|  | B | 809 (9.6) | 395 (13.2) |  | 249 (15.8) | 146 (10.4) |  |  |
|  | O | 3328 (39.6) | 1171 (39.2) |  | 649 (41.2) | 522 (37) |  |  |
| Recipient body mass index, kg/m^2^ | <30 | 6781 (80.8) | 2498 (83.7) | <0.01 | 1334 (84.7) | 1164 (82.6) | 0.11 | 0.058 |
|  | ≥ 30 | 1614 (19.2) | 487 (16.3) |  | 241 (15.3) | 246 (17.4) |  |  |
| Recipient comorbidity number | None | 5249 (62.5) | 2054 (68.8) | <0.01 | 1058 (67.2) | 996 (70.6) | 0.01 | 0.1109 |
|  | 1 | 1931 (23) | 645 (21.6) |  | 343 (21.8) | 302 (21.4) |  |  |
|  | ≥ 2 | 1215 (14.5) | 286 (9.6) |  | 174 (11) | 112 (7.9) |  |  |
| Recipient diabetes ESKD | No | 7454 (88.8) | 2767 (92.7) | <0.01 | 1454 (92.3) | 1313 (93.1) | 0.4 | 0.0309 |
|  | Yes | 941 (11.2) | 218 (7.3) |  | 121 (7.7) | 97 (6.9) |  |  |
| Recipient dialysis | Preemptive transplantation | 848 (10.1) | 174 (5.8) | <0.01 | 83 (5.3) | 91 (6.5) | 0.01 | 0.1111 |
|  | Dialysis < 3 years | 4193 (50) | 950 (31.9) |  | 539 (34.2) | 411 (29.1) |  |  |
|  | Dialysis ≥ 3 years | 3345 (39.9) | 1861 (62.3) |  | 953 (60.5) | 908 (64.4) |  |  |
| Donor age, years | <18 | 294 (3.5) | 36 (1.2) | <0.01 | 15 (1) | 21 (1.5) | 0.06 | 0.0863 |
|  | 18-44 | 1699 (20.2) | 479 (16) |  | 229 (14.5) | 250 (17.7) |  |  |
|  | 45-59 | 2389 (28.5) | 1017 (34.1) |  | 535 (34) | 482 (34.2) |  |  |
|  | 60-69 | 1711 (20.4) | 777 (26) |  | 426 (27) | 351 (24.9) |  |  |
|  | ≥ 70 | 2302 (27.4) | 676 (22.6) |  | 370 (23.5) | 306 (21.7) |  |  |
| Donor blood group | A | 3865 (46) | 1122 (37.6) | <0.01 | 497 (31.6) | 625 (44.3) | <0.01 | 0.2894 |
|  | AB | 378 (4.5) | 35 (1.2) |  | 9 (0.6) | 26 (1.8) |  |  |
|  | B | 794 (9.5) | 171 (5.7) |  | 81 (5.1) | 90 (6.4) |  |  |
|  | O | 3358 (40) | 1657 (55.5) |  | 988 (62.7) | 669 (47.4) |  |  |
|  |  | **Non-sensitized vs HS** | | | **HS AMP vs HS non-AMP** | | | |
|  |  | **Non-sensitized** | **HS** | **P-value** | **HS AMP*** | **HS Non-AMP*** | **P-value** | **Standardized difference** |
| Donor hypertension | No | 5487 (65.4) | 1931 (64.7) | 0.51 | 1005 (63.8) | 926 (65.7) | 0.29 | 0.039 |
|  | Yes | 2908 (34.6) | 1054 (35.3) |  | 570 (36.2) | 484 (34.3) |  |  |
| Donor cause of death | Vascular | 4657 (55.5) | 1726 (57.8) | 0.03 | 907 (57.6) | 819 (58.1) | 0.78 | 0.0101 |
|  | Other | 3738 (44.5) | 1259 (42.2) |  | 668 (42.4) | 591 (41.9) |  |  |
| Donor side graft | Left | 4594 (54.7) | 945 (31.7) | <0.01 | 385 (24.4) | 560 (39.7) | <0.01 | 0.3316 |
|  | Right | 3801 (45.3) | 2040 (68.3) |  | 1190 (75.6) | 850 (60.3) |  |  |
| Donor eGFR, ml/min/1.73 m^2^, mean (SD) |  | 74.8 (27.8) | 75.5 (25.8) | 0,20 | 74.7 (25.1) | 76.4 (26.5) | 0.08 | 0.0633 |
| Graft rank | First transplantation | 7972 (95) | 1348 (45.2) | <0.01 | 779 (49.5) | 569 (40.4) | <0.01 | 0.1838 |
|  | Retransplantation | 423 (5) | 1637 (54.8) |  | 796 (50.5) | 841 (59.6) |  |  |
| Cold ischemia time, hr, mean (SD |  | 14.7 (5.7) | 18.5 (6.2) | <0.01 | 18.9 (6) | 18 (6.4) | <0.01 | 0.1372 |
| Peak cRF | 85-89% |  |  |  | 455 (28.9) | 326 (23.1) | <0.01 | 0.3143 |
|  | 90-94% |  |  |  | 433 (27.5) | 315 (22.3) |  |  |
|  | 95-98% |  |  |  | 471 (29.9) | 407 (28.9) |  |  |
|  | 99-100% |  |  |  | 216 (13.7) | 362 (25.7) |  |  |
| HLA-A mismatches | 0 |  |  |  | 1552 (98.6) | 1008 (71.6) | <0.01 | 0.8309 |
|  | 1 |  |  |  | 19 (1.2) | 308 (21.9) |  |  |
|  | 2 |  |  |  | 3 (0.2) | 92 (6.5) |  |  |
| HLA-B mismatches | 0 |  |  |  | 1531 (98.5) | 915 (65.5) | <0.01 | 0.9402 |
|  | 1 |  |  |  | 21 (1.4) | 349 (25) |  |  |
|  | 2 |  |  |  | 3 (0.2) | 133 (9.5) |  |  |
| HLA-DR mismatches | 0 |  |  |  | 1543 (98.7) | 1103 (79.3) | <0.01 | 0.6751 |
|  | 1 |  |  |  | 20 (1.3) | 253 (18.2) |  |  |
|  | 2 |  |  |  | 0 (0) | 35 (2.5) |  |  |
| HLA-DQβ mismatches | 0 |  |  |  | 1503 (96.3) | 1153 (83.2) | <0.01 | 0.442 |
|  | 1 |  |  |  | 57 (3.7) | 213 (15.4) |  |  |
|  | 2 |  |  |  | 0 (0) | 19 (1.4) |  |  |

*HS: highly sensitized; ESKD:* *End stage kidney disease; HLA: human leukocyte antigen; AMP: acceptable mismatch program
Data are n (%) unless otherwise indicated.
P-values were calculated by chi-squared test for categorical variables and t-test for continuous variables to test the difference between the group and the rest of the study population.*

*Glomerular filtration rate was estimated (eGFR) by using the Modification of Diet in Renal Disease (MDRD) formula (186.3 × (creatinine last observed in µmol/l/88.4)-1.154 × age-0.203 (× 0.742 for women)).*
*HLA mismatch was calculated with acceptable antigen in subtype HLA.*

**Supplementary Figure 9.** **Kidney graft survival with death censored according to sensitization (observation end date: January 1, 2025, Kaplan-Meier method)**

|  | **N** | **Survival at 1 month** | **Survival at 1 year** | **Survival at 5 years** |
| --- | --- | --- | --- | --- |
| **Non sensitized recipients** | 8395 | 96,6% [96,1% - 96,9%] | 94,2% [93,6% - 94,7%] | 87,5% [86,7% - 88,3%] |
| Patients at risk |  | 8008 | 7313 | 3016 |
| **HS AMP recipients** | 1575 | 96,2% [95,2% - 97,1%] | 94,3% [93,0% - 95,3%] | 85,6% [83,4% - 87,5%] |
| Patients at risk |  | 1495 | 1373 | 533 |
| **HS non-AMP recipients** | 1410 | 95,9% [94,7% - 96,8%] | 93,6% [92,1% - 94,7%] | 81,5% [78,9% - 83,8%] |
| Patients at risk |  | 1333 | 1218 | 463 |
| **MS group A** | 5995 | 96,5% [96,0% - 97,0%] | 94,3% [93,7% - 94,9%] | 87,0% [86,0% - 88,0%] |
| Patients at risk* |  | 5693 | 5221 | 2060 |
| **MS group B** | 216 | 95,3% [91,5% - 97,5%] | 93,4% [89,1% - 96,0%] | 84,1% [75,8% - 89,7%] |
| Patients at risk* |  | 202 | 181 | 42 |

HS: highly sensitized

MS group A: moderately sensitized patients with peak cRF <85% and current cRF <85%

MS group B: moderately sensitized patients with peak cRF ≥85% and current cRF ≤70%
[]: 95% Confidence Interval

**Supplementary Figure10.** **Patient survival according to sensitization (observation end date: January 1, 2025, Kaplan-Meier method)**

|  | **N** | **Survival at 1 month** | **Survival at 1 year** | **Survival at 5 years** |
| --- | --- | --- | --- | --- |
| **non sensitized** | 8395 | 99,2% [99,0% - 99,4%] | 95,5% [95,1% - 96,0%] | 80,2% [79,2% - 81,1%] |
| Patients at risk* |  | 8295 | 7841 | 3579 |
| **HS AMP** | 1575 | 99,0% [98,3% - 99,4%] | 95,9% [94,7% - 96,7%] | 82,9% [80,6% - 84,9%] |
| Patients at risk* |  | 1555 | 1483 | 692 |
| **HS non-AMP** | 1410 | 99,0% [98,3% - 99,4%] | 95,0% [93,7% - 96,0%] | 82,8% [80,4% - 84,9%] |
| Patients at risk* |  | 1389 | 1320 | 619 |
| **MS group A** | 5995 | 99,0% [98,7% - 99,2%] | 95,7% [95,2% - 96,2%] | 81,9% [80,7% - 83,0%] |
| Patients at risk* |  | 5898 | 5599 | 2540 |
| **MS group B** | 216 | 99,1% [96,3% - 99,8%] | 94,8% [90,9% - 97,1%] | 77,8% [71,0% - 83,2%] |
| Patients at risk* |  | 212 | 202 | 72 |

HS: highly sensitized

MS group A: moderately sensitized patients with peak cRF <85% and current cRF <85%

MS group B: moderately sensitized patients with peak CRF ≥85% and current cRF ≤70%
[]: 95% Confidence Interval

**Supplementary Figure 11. Forest plot of risk factors for kidney graft failure in the highly sensitized recipient cohort (death not censored) – Cox Model (observation end date: January 1, 2025)**

HR: hazard ratio
eGFR: estimated glomerular filtration rate

**Supplementary Figure 12. Death-censored kidney graft survival in highly sensitized patients eligible for the AMP program according to allocation modality and** **the presence of subsequently delisted preformed DSA (from October 13, 2015 to December 31, 2022)**

|  | **N** | **Survival at 1 month** | **Survival at 1 year** | **Survival at 5 years** |
| --- | --- | --- | --- | --- |
| **HS AMP** | 1575 | 96,2% [95,2% - 97,1%] | 94,3% [93,0% - 95,3%] | 85,6% [83,4% - 87,5%] |
| Patients at risk* |  | 1495 | 1373 | 533 |
| **HS non-AMP without historical DSA** | 1352 | 96,1% [95,0% - 97,0%] | 93,9% [92,5% - 95,1%] | 81,7% [79,0% - 84,0%] |
| Patients at risk* |  | 1283 | 1172 | 435 |
| **HS non-AMP with historical DSA** | 58 | 89,5% [78,0% - 95,1%] | 85,8% [73,6% - 92,6%] | 75,4% [61,2% - 85,0%] |
| Patients at risk* |  | 50 | 46 | 28 |

HS: highly sensitized

[]: 95% Confidence Interval

**Supplementary Table 4. Distribution of acute or chronic rejection among the cause of graft loss in** **highly sensitized (HS) acceptable mismatch program (AMP) recipients and HS non-AMP recipients without DSA and with DSA**

|  | **Acute or chronic rejection cause of graft loss** | | **Other cause of graft loss** | |
| --- | --- | --- | --- | --- |
|  | **Frequency** | **Percent** | **Frequency** | **Percent** |
| HS AMP | 61 | 30% | 143 | 70% |
| HS non AMP without DSA | 81 | 38% | 134 | 62% |
| HS non AMP with DSA | 6 | 43% | 8 | 57% |

*Caution: Acute or chronic rejection data entry is not exhaustive and its quality is not audited.*
